# Wound healing in Atlantic spiny dogfish sharks

**DOI:** 10.1038/s41598-026-67205-6

**Published:** 2026-09-09

**Authors:** Etty Bachar-Wikström, Anthony Anselmo, Aaron R. Thorner, Inga Sidor, Lisa Abbo, Jakob D. Wikström

**Affiliations:** 1 Dermatology and Venereology Division, Department of Medicine (Solna), Karolinska Institutet, 17177 Stockholm, Sweden; 2 Whitman Center, Marine Biological Laboratory, Woods Hole, MA 02543 USA; 3 Center for Cancer Genomics (CCG), Dana-Farber Cancer Institute, Boston, MA 02215 USA; 4 University of New Hampshire, Durham, NH 03824 USA; 5 Dermato-Venereology Clinic, Karolinska University Hospital, 17176 Stockholm, Sweden

**Keywords:** Elasmobranchs, Sharks, Spiny dogfish, Skin, Wound healing, Marine biology, Ecology, Ecology, Ocean sciences, Zoology

## Abstract

Field observations and limited experimental studies indicate that elasmobranchs can repair substantial skin injuries, but the temporal course and cellular composition of wound healing in Atlantic spiny dogfish remain poorly characterized. We conducted an exploratory laboratory study in 20 female Atlantic spiny dogfish (*Squalus acanthias*) using standardized full-thickness skin wounds monitored by serial photography for 35 days, histological analysis at defined post-injury time points, and pooled single-nucleus RNA sequencing of intact and wounded skin. A continuous neoepithelial layer covered all examined wound beds by Day 1, whereas macroscopic wound area decreased progressively over 35 days and dermal denticles remained absent from the repaired surface. Histological examination showed progressive neoepidermal maturation, basement-membrane reformation, collagen deposition, and granulation-tissue organization, indicating that epithelial coverage preceded restoration of normal skin architecture. Single-nucleus RNA sequencing identified epithelial, stromal, vascular, pigment, neural, and immune-cell populations. T and B cells were detected in intact skin, and their relative abundance, together with that of several other leukocyte populations, increased at Day 1 and generally declined by Day 14. Because samples were pooled by time point, these transcriptomic changes are descriptive. These findings characterize rapid early reepithelialization followed by slower tissue remodeling in Atlantic spiny dogfish and provide a foundation for future comparative studies of elasmobranch skin repair.

## Introduction

Hard-to-heal wounds represent a major medical challenge, accounting for a substantial proportion of healthcare expenditure and morbidity^1^. Despite extensive research, relatively few treatments that fundamentally improve tissue regeneration or prevent fibrosis have entered clinical practice. Comparative studies of species with unusual repair capacities can identify biological processes that are difficult to recognize when research is restricted to conventional mammalian models. A prominent example is the axolotl (*Ambystoma mexicanum*), which can regenerate full-thickness skin wounds with restoration of normal tissue architecture and minimal permanent scarring^2,3^. Studies of axolotl wound healing have identified distinctive inflammatory and extracellular-matrix responses, as well as temporal changes in epidermal gene expression, including matrix metalloproteinase-associated responses that may contribute to limiting fibrosis^2,3^. This example **supports** the broader principle that phylogenetically distant vertebrates may reveal conserved or alternative mechanisms of tissue repair, even when they are not direct anatomical models of human skin.

Physiological wound healing typically progresses through three overlapping phases: inflammation, proliferation—including angiogenesis, granulation-tissue formation, and reepithelialization—and remodeling^4^. Broadly corresponding phases have also been described in fishes^5,6^. Key regulatory cell types include platelets or thrombocytes, which initiate inflammatory-cell recruitment and contribute to formation of the provisional extracellular matrix, and macrophages, which modulate the cytokine environment and influence proliferation and wound closure^7^. Neutrophils are recruited rapidly from the circulation and contribute to pathogen clearance and tissue repair^8^, whereas T-cell populations may include both resident and recruited cells that modulate inflammation and support tissue repair^9^. Keratinocytes, fibroblasts, and endothelial cells play central roles in the proliferative phase and restoration of tissue integrity. Rapid reepithelialization may be particularly important in marine species because damaged skin remains continuously exposed to seawater-borne microorganisms and osmotic stress^10^. Although these broad phases are conserved across vertebrates, their timing, cellular composition, inflammatory intensity, extracellular-matrix organization, and final outcomes differ substantially among species. Species-specific characterization is therefore needed before conserved or distinctive mechanisms of repair can be identified.

Sharks and rays experience integumentary injuries throughout their lives as a consequence of mating and conspecific aggression^11,12^, predation^13^, and anthropogenic interactions such as harpooning^14^, entanglement or constriction by marine debris^15,16^, retained fishing gear^17^, tagging, and vessel strikes^18,19^, recently excellently reviewed^20^. Birth-associated umbilical wounds have also been documented in neonatal sharks^18^. In several elasmobranch species, females have substantially thicker skin than males, which has been interpreted as a protective adaptation against mating-associated biting^12,21^. Field observations have documented survival and extensive tissue repair following harpoon injuries^14^, prolonged entanglement^15^, embedded straps^16^, loss of a dorsal fin^22^, and other substantial traumatic injuries in blacktip reef sharks and whale sharks^18,19^. These observations, however, should not be interpreted as evidence that wound healing in sharks is invariably rapid, complete, or uncomplicated. Retained fishing gear may cause tissue necrosis, abscess formation, jaw damage, persistent deformity, morbidity, and death, while pathological studies of elasmobranchs have documented traumatic injury, inflammatory and infectious disease, physiological stress, and emaciation as important contributors to morbidity^23^. Healing rates and outcomes are therefore likely to vary with species, wound type, anatomical location, injury depth, retained foreign material, physiological condition, and environmental factors.

Elasmobranch integument presents a distinctive structural challenge because the skin surface is covered by placoid scales, or dermal denticles, which arise in the dermis and project through the epidermis^24,25^. In experimental skin wounds in nurse and leopard sharks, wound contraction and reepithelialization preceded restoration of the denticulate surface, with extensive replacement of dermal denticles requiring approximately four months^26^. Histological examination of naturally occurring wounds in a free-ranging blacktip shark similarly revealed epidermal hyperplasia, granulation tissue, fibrosis, skeletal-muscle injury and regeneration, and an absence of denticles within the healing tissue^27^. Standardized punch-wound studies in yellow stingrays further demonstrate that controlled longitudinal investigation of wound healing is feasible in elasmobranchs^28^. Shark skin is also covered by a relatively thin but molecularly distinctive mucus layer^29^, and studies of blacktip reef sharks have found broadly similar bacterial and fungal communities on wounded and apparently healthy skin^30,31^. Together, frequent exposure to trauma, continuous contact with the aquatic environment, distinctive denticulate integument, and capacity for substantial but variable tissue repair make marine elasmobranchs informative comparative systems for investigating vertebrate wound healing.

Elasmobranchs occupy an important evolutionary position in vertebrate immunology because they represent one of the earliest-diverging extant jawed vertebrate lineages with an adaptive immune system based on somatically rearranged antigen receptors. Sharks exhibit antibody affinity maturation and immunological memory^32^ and produce IgNAR, a heavy-chain-only immunoglobulin that is structurally distinct from conventional vertebrate antibodies^33^. Immunoglobulin isotypes have also been detected in spiny dogfish skin mucus^34^, indicating that adaptive immune components are present at the cutaneous surface and may contribute to barrier defence. However, little is known about the composition and temporal dynamics of lymphocyte populations during elasmobranch wound repair. Characterizing the relative immune-cell composition of intact and wounded skin may therefore provide insight into cellular responses associated with tissue repair in this evolutionarily distinctive vertebrate lineage.

The Atlantic spiny dogfish (*Squalus acanthias*) is a cold-temperate elasmobranch that can be maintained under controlled aquatic conditions and is sufficiently large for standardized full-thickness wounding, serial photography, and collection of tissue for histological and molecular analyses. These characteristics make it experimentally suitable for longitudinal investigation of cutaneous repair. Its skin combines structural features shared with other vertebrates, including type I collagen^35^, with distinctive elasmobranch features such as placoid scales, or dermal denticles^24,25^, and a relatively thin mucus layer whose molecular composition differs from that of teleost fishes^29^. These similarities do not make shark skin a direct anatomical equivalent of mammalian skin, and its distinctive structural features may influence epithelial migration, barrier restoration, inflammation, and restoration of the normal skin surface following injury. Characterizing wound repair directly in this species is therefore necessary before its responses can be meaningfully compared with those of other vertebrates. Accordingly, the primary objective of this exploratory study was to characterize the temporal and cellular course of cutaneous wound healing in Atlantic spiny dogfish following standardized full-thickness skin injury. We combined serial photography, histological analysis, and single-nucleus RNA sequencing to examine macroscopic wound closure, reepithelialization and tissue remodeling, and changes in the relative cellular composition of intact and wounded skin. This characterization provides a foundation for future comparative investigations of conserved and lineage-specific features of vertebrate wound repair.

## Materials and methods

### Animals

Twenty female Atlantic spiny dogfish (*Squalus acanthias*) were obtained in 2022 from commercial hook-and-line fishers in Chatham, Massachusetts, USA. Only female sharks were available, likely because the commercial fishery predominantly targets female schools^36^. The sharks were housed in single-species groups at the Marine Resources Center, Marine Biological Laboratory, Woods Hole, Massachusetts, in a natural-seawater flow-through system maintained at 14 °C. Animals were acclimatized for one week before the experimental procedures and were fed three times per week with food-grade frozen capelin (Atlantic-Pacific, North Kingstown, RI, USA) and freshly frozen, locally caught squid. All experimental procedures were approved by the Institutional Animal Care and Use Committee of the Marine Biological Laboratory (protocol no. 22-22) and were conducted in accordance with relevant institutional guidelines and regulations.

### Skin sampling

For each biopsy procedure, sharks were gently captured using a net and transferred individually to a covered 90-L procedure tank containing seawater supplemented with the general anesthetic AQUI-S^®^ 20E (eugenol) at a concentration of 37.5 mg/L under INAD no. 11-741. During the initial procedure, each shark was tagged on the dorsal fin with a plastic identification tag (Floy Tag Inc., Seattle, WA, USA). Six full-thickness skin biopsies, 4 mm in diameter, were collected from each shark using a 4-mm punch biopsy instrument (Kai Medical, Solingen, Germany). Biopsies were distributed across the ventral and bilateral dorsolateral skin regions. The excised specimens constituted intact baseline skin samples collected on Day 0, while the resulting 4-mm biopsy defects constituted the experimental wounds. This procedure generated six wounds per shark and 120 wounds in total.

Follow-up sampling was performed on Days 1, 14, and 35 post-injury. At each time point, two wounds per shark were excised using a 6-mm punch biopsy instrument centered on the original wound, thereby including the healing wound and adjacent tissue. The resulting 6-mm biopsy sites were closed with absorbable Vicryl sutures (Ethicon Inc.). The sampling protocol therefore generated intact baseline skin samples from Day 0 and healing wound samples from Days 1, 14, and 35. At the end of the study, the sharks were euthanized by prolonged immersion in seawater containing AQUI-S® 20E at 37.5 mg/L.

### Wound assessment by histology

Skin biopsy specimens were fixed in 4% formaldehyde (Fisher Scientific) and processed by routine paraffin embedding and sectioning at the ZooQuatic Laboratory (Durham, NH, USA). Sections were stained with hematoxylin and eosin (H&E) to assess general tissue morphology, Masson’s trichrome (MT) to evaluate collagen deposition and tissue organization, periodic acid–Schiff–Alcian blue (PAS–AB) to visualize mucin-containing cells and the cutaneous mucus layer, and Gram stain to assess bacterial presence and Gram reaction. For each biopsy type and stain, six sections were examined.

Reepithelialization was defined as the presence of a continuous epithelial cell layer covering the wound bed. A blinded quantitative assessment of reepithelialization was initially planned. However, because all examined wounds showed complete epithelial coverage at Day 1, percentage reepithelialization could not distinguish among samples. Reepithelialization and other histological features were therefore assessed qualitatively in a non-blinded manner. No inferential statistical analyses were performed on the histological observations.

### Wound assessment by photography

Each of the six wounds per shark was monitored by serial photography using an iPhone 13 Pro (Apple Inc.). A standard ruler was positioned adjacent to the wounds in each image to provide a spatial scale. Images were analyzed using ImageJ software (National Institutes of Health, Bethesda, MD, USA). For each image, a known distance on the ruler was measured using the Straight Line tool and used to calibrate the spatial scale. The visible wound margin was then traced manually using the Freehand Selections tool, and the enclosed wound area was recorded in mm^2^. Measurements were entered into Microsoft Excel and used to follow changes in macroscopic wound area over time. All six wounds from each shark were followed photographically for up to 35 days.

### Single nucleus RNA sequencing (snRNA-seq)

Nuclei were isolated from fresh-frozen skin biopsy specimens using a protocol adapted from Slyper et al.^37^. Low-retention microcentrifuge tubes (Fisher Scientific, Hampton, NH, USA) were used throughout the procedure to minimize nuclei loss. Tissues were manually dissociated by chopping with fine spring scissors for 10 min in TST solution containing 146 mM NaCl, 10 mM Tris–HCl (pH 7.5), 1 mM CaCl₂, 21 mM MgCl₂, 0.03% Tween-20, and 0.01% BSA, prepared as described by Slyper et al.^37^. The tissue suspension was homogenized, filtered through a 30-µm MACS SmartStrainer (Miltenyi Biotec, Bergisch Gladbach, Germany), and centrifuged at 500 × *g* for 10 min at 4 °C. The resulting nuclei pellet was resuspended in 100 µL of 0.1 × lysis buffer containing 10 mM Tris–HCl (pH 7.4), 10 mM NaCl, 3 mM MgCl₂, 1% BSA, 1 mM DTT, 1 U/µL RNase inhibitor, 0.01% Tween-20, 0.01% NP-40, and 0.001% digitonin and incubated on ice for 2 min, following the 10 × Genomics nuclei-permeabilization protocol (CG000375). The nuclei were then centrifuged again at 500 × *g* for 10 min at 4 °C. The final nuclei pellet was resuspended in 150 µL of 1 × Diluted Nuclei Buffer prepared from 20 × Nuclei Buffer (10 × Genomics, PN-2000207) supplemented with 1 mM DTT and 1 U/µL RNase inhibitor. Nuclei were stained with trypan blue and counted manually using an INCYTO C-Chip Neubauer Improved disposable hemocytometer (VWR International, Radnor, PA, USA). Samples were pooled by time point: eight Day 0 biopsy samples were combined into one pool, and four biopsy samples were combined into one pool at each of Days 1, 14, and 35. The Day 35 pool subsequently failed quality-control criteria and was excluded from downstream analyses. Up to 10,000 nuclei from each pooled sample were loaded into one channel of a Chromium Next GEM Chip K and processed using the Chromium Controller (10 × Genomics, Pleasanton, CA, USA). Barcoded cDNA generation and library construction were performed according to the manufacturer’s instructions using the Chromium Next GEM Single Cell 5′ Reagent Kit v2 (User Guide, Revision E). Libraries were normalized, pooled, and sequenced on an Illumina NovaSeq 6000 using an SP100 flow cell (Illumina, San Diego, CA, USA), with 26 cycles for Read 1, 10 cycles for Index 1, 10 cycles for Index 2, and 90 cycles for Read 2.

### Bioinformatic analysis of snRNA-seq data

Sequencing data were demultiplexed and converted to FASTQ files using the Cell Ranger mkfastq pipeline (10 × Genomics). Reads were aligned to the Atlantic spiny dogfish reference genome assembly GCA_030390025.1 (ASM3039002v1; scaffolds 1–30), and gene–barcode count matrices were generated using the Cell Ranger count pipeline with STAR v2.7.2a.

Ambient RNA background was removed from the raw Cell Ranger gene-expression matrices using CellBender remove-background, which was run for 150 epochs for each pooled sample. The numbers of nuclei estimated by Cell Ranger before quality-control filtering and retained for downstream analysis are summarized in Table 1. The Day 35 sample did not pass the quality-control criteria and was excluded from downstream analyses.

**Table 1:** Nuclei counts before and after quality-control filtering. Numbers of nuclei estimated by Cell Ranger before quality-control filtering and retained for downstream analysis for each pooled time-point sample.

Sample/time point	Estimated nuclei before QC (Cell Ranger)	Nuclei after QC, used for analysis
D0	9115	8261
D1	5591	4876
D14	15,466	7938
D35	4691	0*

*The Day 35 sample did not pass the quality-control criteria and was excluded from downstream analyses.

CellBender-filtered count matrices were analyzed using Scanpy, and the analysis workflows were recorded in Jupyter notebooks. Nuclei with fewer than 200 detected genes were excluded, genes detected in fewer than three nuclei were removed, and nuclei in which mitochondrial transcripts accounted for 20% or more of total transcript counts were excluded. Predicted doublets were identified and removed using Scrublet. Uniform Manifold Approximation and Projection was used for dimensionality reduction and visualization of the retained nuclei.

Expression data from the retained samples were integrated using Harmony to reduce sample-specific variation while preserving biological variation. Cell clusters were annotated using the expression of cell-type marker genes together with functional gene-set enrichment analysis.

### Secondary analysis of published zebrafish scRNA-seq data

To provide a comparative vertebrate context for interpreting the cellular composition of Atlantic spiny dogfish skin, cell-type proportions were quantified in a published zebrafish skin scRNA-seq dataset. Metadata were obtained from the dataset published by Jiang et al.^38^, which profiled more than 250,000 cells from 18 zebrafish tissues using Microwell-seq. Per-cell metadata were extracted from the worksheet titled “cell barcode of each cell” in Supplementary Data Sheet 2 and included cell barcodes, cluster identifiers, tissue labels, and assigned cell-type annotations. Cells associated with skin tissue were retained, and the “Annotation” field was used as the assigned cell type. Cell counts were aggregated by annotation using Python v3.10 and the pandas library, and the relative proportion of each cell type was calculated by dividing its count by the total number of retained skin cells. No reclustering or reannotation was performed. This analysis therefore provided a descriptive summary of the cell-type composition reported for zebrafish skin in the original dataset.

## Results and discussion

### Project overview

**Fig. 1:**
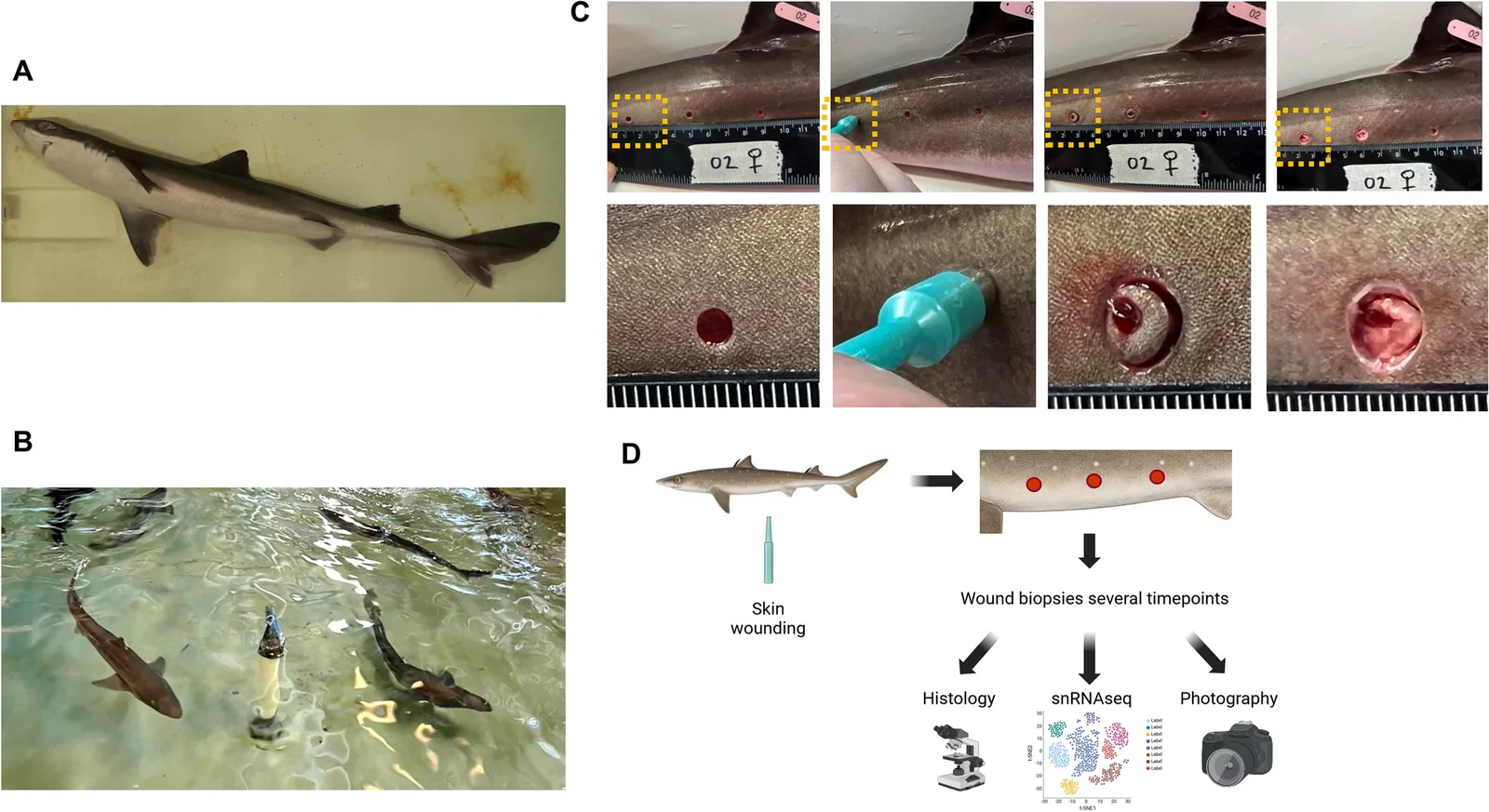
Species studied and project overview. (**A**, **B**) Atlantic spiny dogfish sharks (*Squalus acanthias*), a widespread and abundant shark species, were captured from the wild and housed in saltwater pools. (**C**) Wound harvest technique. At day 0, 4 mm punches were harvested (6 per shark, not shown) followed by two 6 mm punches per time point to harvest the wounds at days 1, 14 and 35 (biopsy at day 1 shown, note that the 4 mm wound is excised with a 6 mm punch tool). Size: 1 mm between lines. (**D**) Wound healing was monitored by serial photography, and biopsy samples from different time points were analyzed by histology and single-nucleus RNA sequencing (snRNA-seq) to study wound healing speed and to characterize cell types. In total 20 animals were used.

Figure 1 summarizes the experimental design and analytical workflow used to characterize wound healing in Atlantic spiny dogfish. Wound repair was monitored longitudinally by serial photography and assessed at defined post-injury time points using histological and single-nucleus RNA-sequencing analyses. These complementary approaches were used to examine macroscopic wound closure, tissue-level repair, and changes in the cellular composition of intact and wounded skin.

### Intact skin histology

**Fig. 2:**
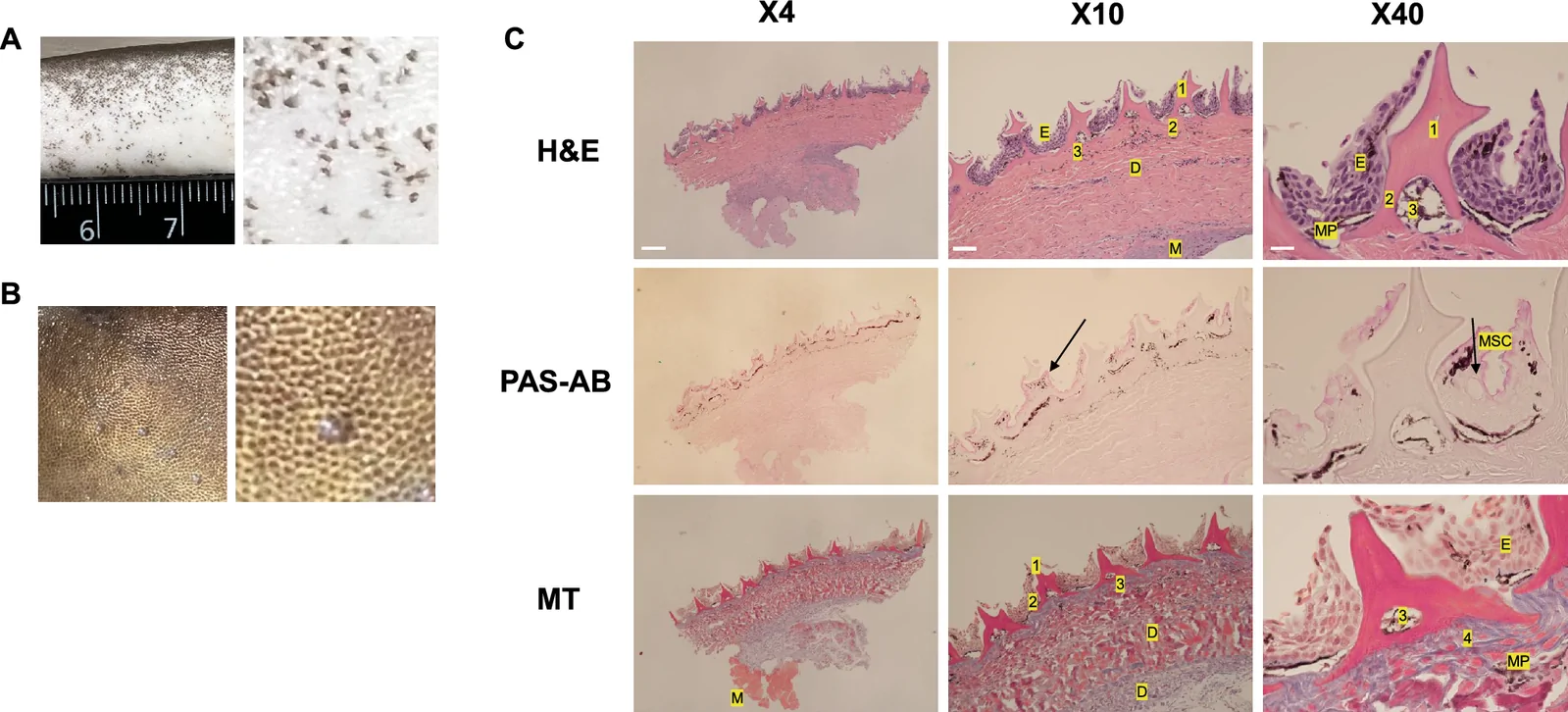
(**A**, **B**) Spiny dogfish skin. (**A**, **B**) Skin photos. The skin of the spiny dogfish ranges in color from grey to white to brown and is covered with scale-like structures known as denticles. (**C**) Histology. Sagittal sections of one biopsy are shown. Hematoxylin–Eosin (H&E) staining shows the different skin layers as indicated: epidermis (E), dermis (D) and denticles (1,2). Masson-Trichrome (MT) staining shows a more refined division of the skin layers and allows to differentiate hard tissues (e.g. denticles) from soft ones. Layers are indicated: epidermis E, dermis (D) muscle (M) melanin pigmentation (MP), mucin secretory cells (MSC), denticles (1,2). Denticles consist of a backward-pointing spine (1), a basal plate covered with enamel (2) and a pulp cavity (3). Blue (4) indicates collagen. PAS–AB staining shows the mucosal layer in magenta-pink at the apical part of the epidermis and both HE and PAS staining shows the location of the mucin vacuoles throughout the epidermis layer. Size bars: 250 μm (4x), 100 μm (10X) and 25 μm (40X).

Histological examination of intact Atlantic spiny dogfish skin showed an epidermis and underlying dermis covered by backward-pointing dermal denticles (Fig. 2). At higher magnification, epithelial cells, including keratinocytes and melanocytes, were observed between the denticles. PAS–AB staining highlighted a thin mucosal layer along the apical surface of the epidermis and mucin-containing vacuoles distributed throughout the epidermal layer. These findings were consistent with previous descriptions of shark integument^24,25^. Our previous mass-spectrometry analysis further indicated that the mucin layer of Atlantic spiny dogfish differs from that of teleost fishes in both thickness and molecular composition^28^.

### Wounded skin histology

**Fig. 3:**
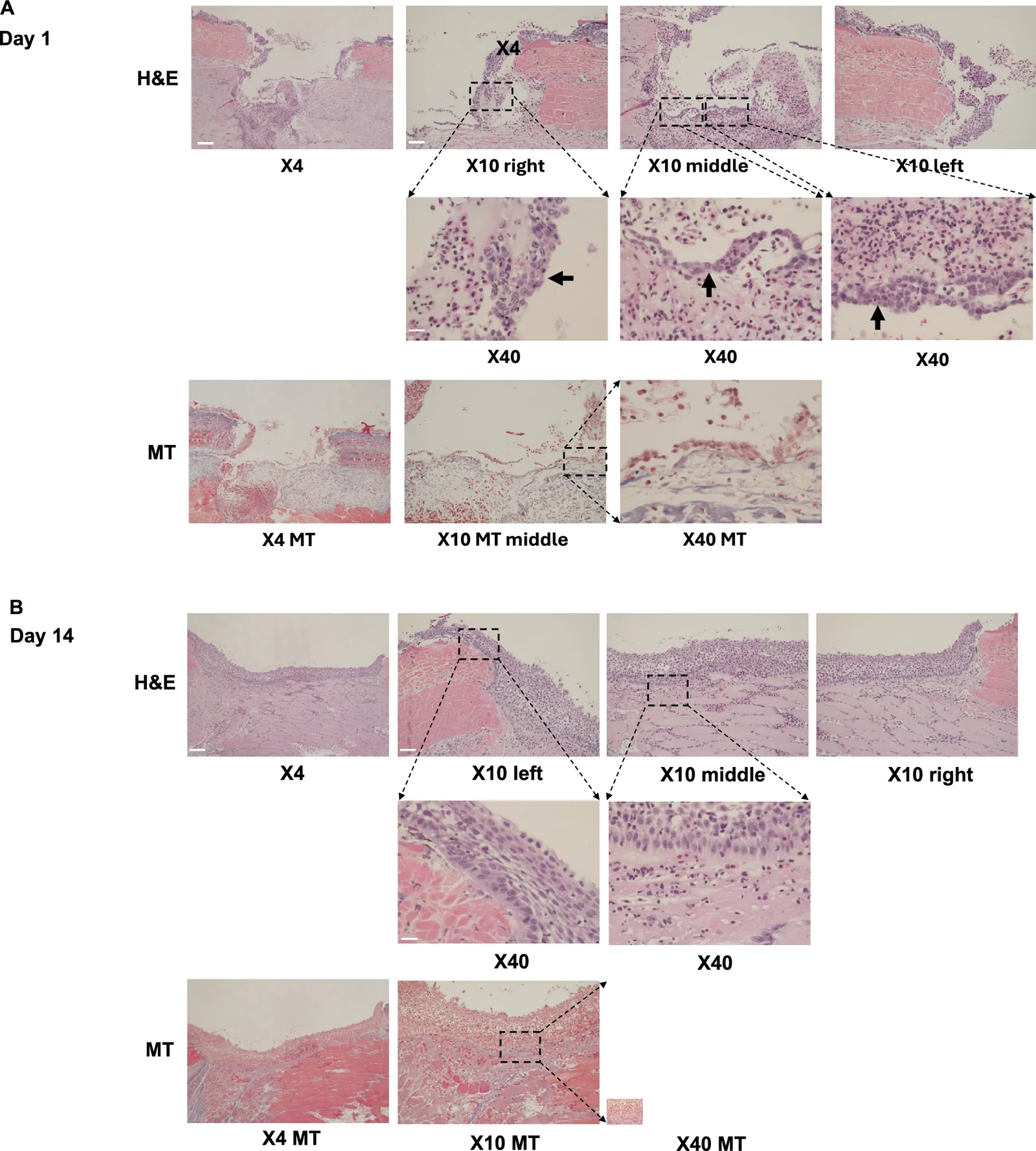

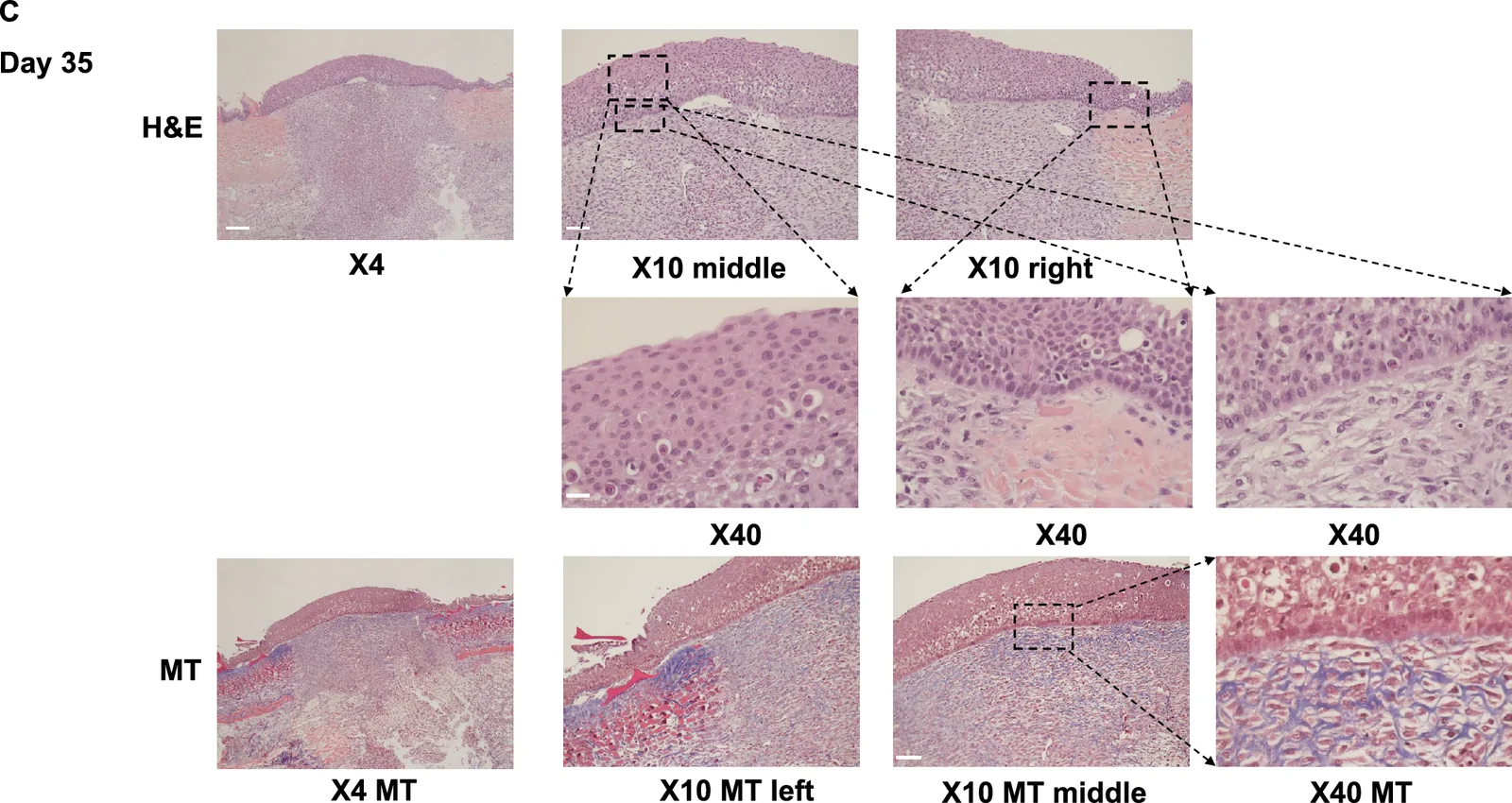
Histological wound healing. Representative images. (**A**) Day 1 after wounding. Wounds are full thickness to the muscle and basal membrane is absent. Note that keratinocytes migrate directly on a thin collagen layer on the muscle before any granulation tissue has developed and has fully reepithelialized the wound by 24 h. Arrows indicate neoepidermis. Erythrocytes and heterophils are present. (**B**) Day 14 after wounding. The scar is covered by a thick stratified epidermis with some leukocytes and intercellular edema still present in the epidermis and dermis. Dermal regeneration has started with visible collagen in the MT staining (blue color) however the wound is still depressed which matches the macroscopic healing process (Fig. 4). (**C**) Day 35 after wounding. The scar is not depressed and is covered by a thick stratified epidermis (hyperplasia). A few apopotic cells (likely involution). Dense bed of well-organized granulation tissue, still a defect in the thick fibers of the compact dermis, but clear collagen deposition in maturing granulation tissue on MT. Prominent exocytosis of heterophils. Note that denticles are still absent. H&E = hematoxylin-eusin, MT = Masson-Trichrome. Size bars: 250 μm (4x), 100 μm (10X)) and 25 μm (40X). N = 40 wounds from 20 fishes in total.

**Fig. 4:**
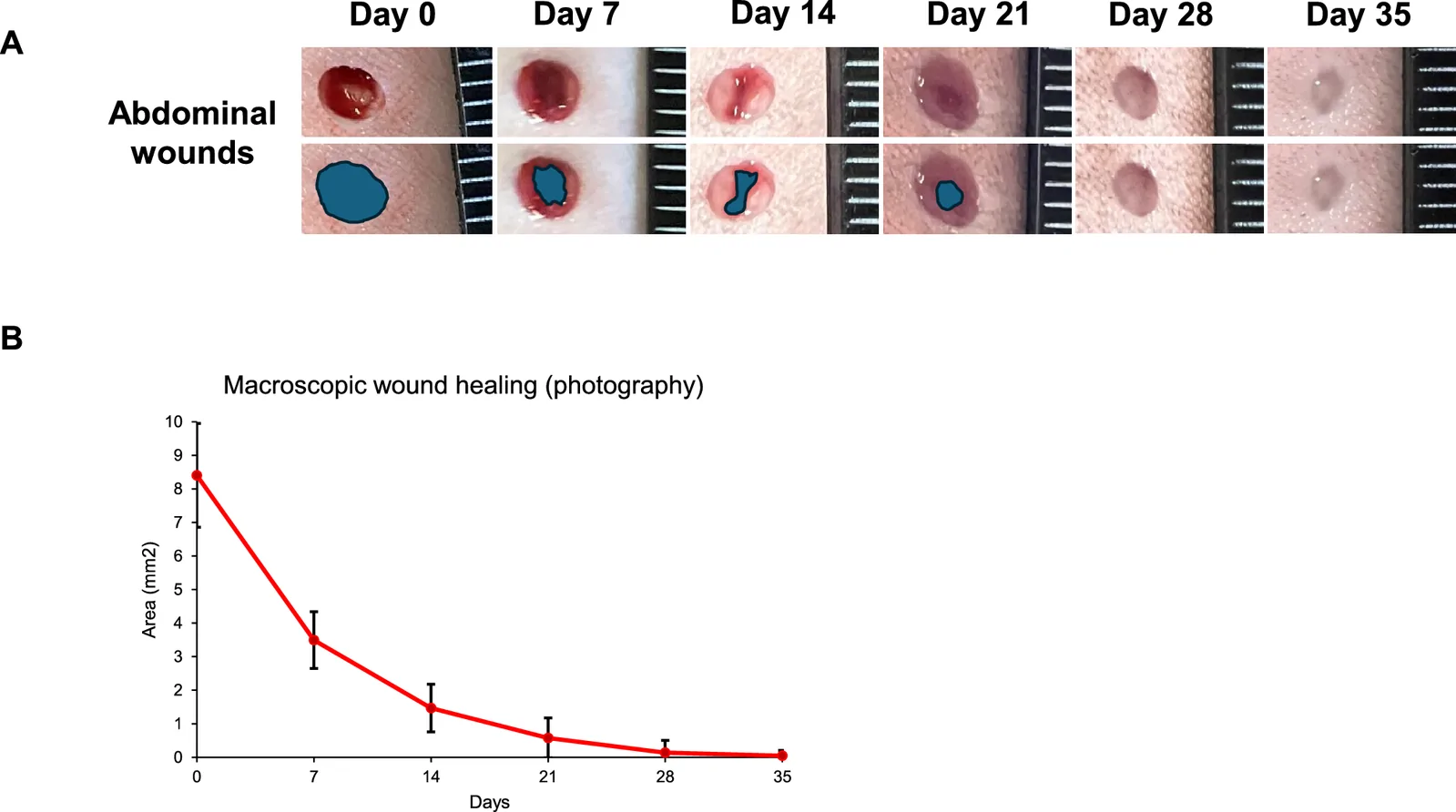
(**A**, **B**) Macroscopic wound healing. Macroscopic healing and skin regeneration as documented by serial photography. (**A**) Wounds are indicated with blue color in the lower row. Note that reepithelialization precedes macroscopic healing as demonstrated histologically in Fig. 3A. By Day 35 denticles are still absent in the scars. Size: 1 mm between lines. (**B**) Quantification of serial photography N = 120 wounds at start from a total of 20 fishes (Day 0 = 120, Day 7 = 80, Day 14 = 80, Day 21 = 40, Day 28 = 40, Day 35 = 40). Each wound was imaged at the indicated time points. Error bars indicate standard deviation. No comparative statistical analysis were performed.

**Fig. 5:**
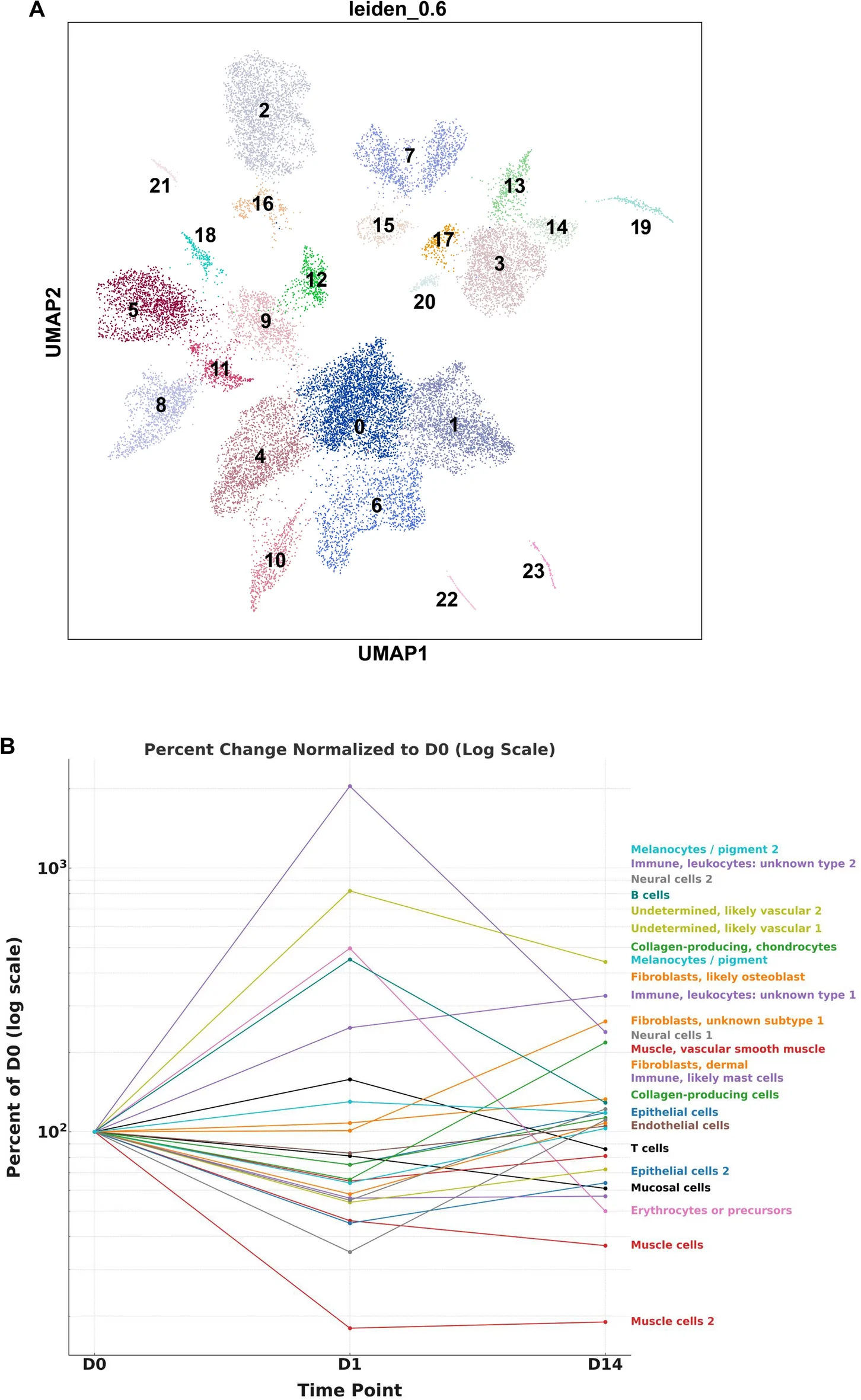
Clustering and marker gene identification from single-nucleus RNA-seq data in wound healing. (**A**) UMAP plot displaying transcriptionally distinct clusters (0–23) derived from skin biopsy nuclei (Day 0, intact skin). Each point represents a nucleus, colored by cluster identity (Table 2). (**B**) Change in cell type composition during wound healing (Day 0, Day 1 and Day 14 after wounding). Colors indicate biological categories of cell types (e.g., blue for epithelial, red for muscle, purple for immune). Labels are positioned at D14 to show final values, and the y-axis is log-scaled to display wide-ranging changes relative to D0. The graph was generated from Table 3. The snRNA-seq analysis included 20 skin biopsies from four sharks: 8 intact skin samples (two biopsies per shark) and 4 wound samples at each post-injury time point (Days 1, 14, and 35). Samples were pooled by time point: eight Day 0 biopsy samples were combined into one pool, and four biopsy samples were combined into one pool at each of Days 1 and 14.

Remarkably, just one day post-wounding, a newly formed epithelial layer of migrating keratinocytes was already covering the wound bed of all samples and made measurements of reepithelialization impossible nor needed (Fig. 3A). These epidermal cells migrate along a very thin collagen anchor layer, as seen in MT staining. This early reepithelialization is not discernible in macroscopic photographs (Fig. 4). Reepithelialization was a qualitative assessment defined as when a thin layer of keratinocytes was covering the wound bed. Within the wound bed, numerous ovoid to flattened erythrocytes accumulate, indicating bleeding from the punch biopsy and aligning with the snRNA-seq data (Fig. 5B and Table 3). These erythrocytes exhibit abundant eosinophilic cytoplasm, centrally located oval nuclei, and dense chromatin. They are embedded in fibrin coagula, accompanied by smaller numbers of intact and lysed neutrophils/granulocytes, which display limited eosinophilic cytoplasmic granules and round, segmented eccentric nuclei. Further, PAS staining shows that by day one post-wounding, a mucus layer begins to form at the edge of the developing neoepidermis, with mucus-filled MSCs visible. The basement membrane is distinct on the lateral sides of the wound bed, marked by pigmentation, but remains absent beneath the neoepidermis. No bacteria were observed in the examined sections by Gram staining (Supp Fig. 1A).

**Table 2:** Putative cell types identified by snRNA-seq (see Fig. 5A).

Cluster	Putative cell type	Key genes
0	Epithelial cells (krt18+)	krt18, krt8, tgm2, ppl, fgfr2
1	Collagen-producing cells	col4a1, col4a6, col18a1, col4a2
2	Muscle cells (skeletal, tnnt2+)	neb, obscn, tnnt2, myh7b
3	Fibroblasts, unknown subtype 1	col1a2, col1a1, col5a2, col12a1
4	Mucosal cells (krt8++)	muc4, muc19, krt8, krt18, tgm2
5	T cells	cd3e, cd3g, ptprc, dock10
6	Epithelial cells	gmds, krt8, nhs, ppl
7	Endothelial cells	flt1, prex2, ptprb, alcam
8	Immune, leukocytes: unknown type 1	dock2, ptprc, arhgap15, fmnl3
9	Erythrocytes or precursors	hbb, epb41, slc4a2, sema7a
10	Immune, likely mast cells	adgrf5, kit, alcam, dclk2
11	Immune, leukocytes: unknown type 2	ptprc, mmp9, itgb2, ankrd44
12	Undetermined, likely vascular	pde4b, abca2, vegfc, zeb2
13	Fibroblasts, dermal	col7a1, col1a2, lama2, col5a2
14	Fibroblasts, likely osteoblast	col12a1, thbs2, col1a2, eln
15	Muscle, vascular smooth muscle	acta2, myh11
16	Muscle cells (skeletal, tnnt3+)	neb, tnnt3, obscn, myh4
17	Collagen-producing, chondrocytes	col2a1, col12a1, col1a2, col1a1, fndc1
18	B cells	blnk, pax5, ptprc, arhgap45, btk
19	Melanocytes (mlph+, tyrp1+)/pigment	mlph, tyrp1, slc24a5, pmel
20	Neural cells 1	nrxn1, unc5d, nav1, sema6b
21	Undetermined, likely vascular	ank2, col4a1, col18a1, prkd1
22	Melanocytes (mlph+, qnr-71+)/pigment	mlph, qnr-71, ltk, kcnma1, nrxn1, pcdh11x
23	Neural cells 2	dgkb, kcnma1, nrxn1, cacna1h

**Table 3:** Cell types changes during wound healing identified by snRNA-seq (see Fig. 5B). D0 = Day 0 (day of wounding); D1 = Day 1 after wounding; D14 = Day 14 after wounding.

Cluster	Putative Cell Type	D0	D1	D14	D0	D1	D14	D0	D1	D14
0	Epithelial cells	1366	604	1548	1654	1239	1950	100	75	118
2	Muscle cells	1348	364	480	1632	747	605	100	46	37
1	Collagen-producing cells	918	405	999	1111	831	1259	100	75	113
4	Mucosal cells	868	414	511	1051	849	644	100	81	61
6	Epithelial cells	660	174	404	799	357	509	100	45	64
5	T cells	555	516	458	672	1058	577	100	158	86
3	Fibroblasts, unknown subtype 1	448	267	1130	542	548	1424	100	101	262
7	Endothelial cells	444	218	446	537	447	562	100	83	105
10	Immune, likely mast cells	274	90	149	332	185	188	100	56	57
8	Immune, leukocytes: unknown type 1	194	284	611	235	582	770	100	248	328
9	Erythrocytes or precursors	191	559	91	231	1146	115	100	496	50
16	Muscle cells	178	19	32	215	39	40	100	18	19
13	Fibroblasts, dermal	138	88	176	167	180	222	100	108	133
15	Muscle, vascular smooth muscle	131	50	102	159	103	128	100	65	81
14	Fibroblasts, likely osteoblast	126	43	131	153	88	165	100	58	108
19	Melanocytes/pigment	72	27	71	87	55	89	100	64	103
17	Collagen-producing, chondrocytes	62	24	130	75	49	164	100	66	218
20	Neural cells 1	58	12	62	70	25	78	100	35	111
21	Undetermined, likely vascular	56	18	39	68	37	49	100	54	72
12	Undetermined, likely vascular	42	203	178	51	416	224	100	819	441
18	B cells	38	101	47	46	207	59	100	450	129
23	Neural cells 2	34	11	40	41	23	50	100	55	122
11	Immune, leukocytes: unknown type 2	30	362	69	36	742	87	100	2044	239
22	Melanocytes/pigment	30	23	34	36	47	43	100	130	118
	Total number of cells	8261	4876	7938	10,000	10,000	10,000			

By day 14 post-wounding, the neoepidermis has thickened significantly, exceeding the surrounding intact skin in depth (Fig. 3B). Again, this progression remains unapparent in macroscopic images, where the wounds appear smaller but not fully healed (Fig. 4). Keratinocytes within the neoepidermis have now differentiated, forming several epidermal layers starting from a basal keratinocyte layer. The basement membrane has begun to reform beneath this layer. Some inflammatory cells persist, with mild leukocyte exocytosis and intracellular edema evident in the healing epidermis. MT staining reveals a thin collagen layer beneath the neoepidermis (blue), as well as an organized fibrin clot layer beneath the epidermis. PAS staining is more intense than day one, confirming mucin presence within the neoepidermis, particularly in its apical region (Supp Fig. 1B).

By day 35 post-wounding, epidermal hyperplasia and dermal fibrosis are clearly visible (Fig. 3C). The epidermis has further thickened, and the dermis shows dense collagen deposition, resembling maturing scar tissue. This histological impression now matches the macroscopic photos in which a scar is seen on day 35 (Fig. 4). Prominent leukocyte exocytosis persists. Some apoptotic epithelial cells are observed, possibly indicating the onset of epidermal hyperplasia regression. Denticles remain absent. Although histological shark wound healing studies are scarce, a case report on a tropical black tip shark exhibiting large and small skin wounds showed similar epidermal hypertrophy as well as absence of denticles in healing wounds (wound duration was unknown)^27^ although further comparisons are difficult to make.

The rapid reepithelialization observed within 24 h is comparable to that seen in adult zebrafish (*Danio rerio*)^39^, a well-studied model for wound healing in bony fish. Zebrafish, however, are tropical warm-water species with optimal temperatures between 26 °C and 28.5 °C, while spiny dogfish are adapted to cold-water environments with optimal temperatures between 7 °C and 15 °C. Given that metabolic processes and cellular activity generally slow in colder temperatures, the speed of reepithelialization in spiny dogfish is striking. This suggests that cold-water elasmobranchs may possess adaptations that allow for rapid epithelial closure despite reduced metabolic rates.

In salmonids, reepithelialization requires ~ 3 days under similar temperatures^5^, underscoring that dogfish reepithelialization is unusually rapid compared with cold-water actinopterygians. This observation raises the possibility that elasmobranchs may have evolved unique epithelial migration or signaling mechanisms, perhaps involving enhanced keratinocyte motility or pre-existing immune-epithelial crosstalk that promotes faster closure. Such rapid reepithelialization may serve an adaptive function, providing early physical protection against seawater-borne pathogens, mechanical abrasion, and osmotic stress in a marine environment^10^. The early presence of mucus-secreting cells further supports this idea, as mucus layers can serve antimicrobial and barrier roles before the full restoration of epidermal structure.

Although challenging to test experimentally, it is tempting to hypothesize that tropical elasmobranchs—living in warmer environments with higher pathogen loads and faster metabolic rates—might exhibit even faster wound closure^14–16,19^. In support of this idea, a photography study on tagging induced umbilical wounds (or scars) in black tip sharks (*Carcharhinus melanopterus*) showed significant healing after 6 days and more or less complete healing within 24 days^18^. Similar photography based healing times of 22 days were found after induction of 8 mm punch wounds in tropical yellow stingrays (*Urobatis jamaicensis*)^28^. Although not directly relevant here, other black tip reef shark studies showed that sharks with traumatic skin wounds acquired in the wild do not differ in their skin bacterial microbiome^30^ nor in their fungal mycobiomes^31^. Future comparative studies across temperature gradients and species may reveal whether rapid epithelial regeneration is a general feature of sharks or a particularly refined trait in cold-water species such as the spiny dogfish.

### Single nucleus RNA-sequencing – cell type dynamics during wound healing

Wound healing is a multifaceted process involving numerous cell types, many of which are difficult to identify through histology alone. To gain a more comprehensive view of cellular composition in intact and wounded shark skin, we performed single-nucleus RNA sequencing (snRNA-seq). Sequencing reads were aligned to the available reference genomes of spiny dogfish (*Squalus acanthias*). An important limitation is that changes in snRNA-seq cell proportions may reflect true immune-cell recruitment, but they may also be influenced by tissue sampling, nuclei recovery, or relative changes in other cell populations and thus results should be interpreted with caution.

In intact skin (Day 0 samples), epithelial cells—likely keratinocytes—were the most abundant cell type, followed by collagen-producing cells (presumably fibroblasts) and mucosal cells (likely mucin-secreting secretory cells) (Fig. 5A, Tables 2, 3), consistent with histological findings (Fig. 2C). T-cells were notably prevalent, even more common than endothelial cells, suggesting a substantial reservoir of resident T-cells. Additional immune-related populations, including mast cells and two subtypes of uncharacterized leukocytes, further support the hypothesis that shark skin is immunologically enriched.

Skeletal muscle cells were also highly represented, likely reflecting the deep nature of the punch biopsies. Two distinct melanocyte populations were identified, presumably contributing to pigmentation and UV protection. A small cluster expressing cartilage-associated markers, suggestive of chondrocytes and proximity to underlying cartilaginous structures of the endoskeleton, was detected, although its precise identity remains uncertain.

Comparative reanalysis of existing zebrafish skin snRNA-seq data^38^ revealed several differences (Supp Table 1). In zebrafish, mucosal cells are the most abundant skin cell type, whereas they rank fourth in spiny dogfish—consistent with the observation that sharks are less "slimy" than bony fish (authors' personal observation and^29^). Innate immune cells rank as the eighth most abundant population in zebrafish, and T-cells were not reported, although another study estimated that T-cells comprise 16.9% of zebrafish skin lymphocytes^40^. These interspecies comparisons should be interpreted cautiously, as species differences as well as methodological differences including sequencing method, tissue processing, and annotation strategy may introduce bias.

In wounded spiny dogfish skin, we observed a pronounced spike in immune cells—particularly B-cells (potentially secreting various immunoglobulins including shark-specific IgNAR), T-cells, and unclassified leukocyte populations—on Day 1 post-wounding (Fig. 5B, Table 3). By Day 14, most of these immune cell populations had returned to near-baseline levels, suggesting a tightly regulated but rapid immune response. Macrophages were notably absent from the dataset, possibly due to limitations in marker resolution or differences in elasmobranch immune cell types.

As expected, erythrocyte numbers increased on Day 1, likely reflecting post-biopsy bleeding. Concurrently, epithelial cell numbers decreased due to epidermal removal at the wound site. Collagen-producing cells and fibroblasts showed a variable increase consistent with scar tissue remodeling, while dermal fibroblast populations remained stable, potentially reflecting subtype-specific roles in wound repair. Mucosal cells remained reduced through Day 14, suggesting that full restoration of glandular skin features lags structural repair. Skeletal muscle cells were reduced by more than 50% on Day 1, a likely result of physical tissue removal, with no significant regeneration observed by Day 14. Two neural cell types declined initially but recovered by Day 14, indicating rapid peripheral nerve regeneration. Most endothelial cells remained unchanged, though two unknown vascular-associated cell types increased significantly, suggesting a possible expansion of specialized vasculature components during wound healing.

Our snRNA-seq results reveal that immune cell recruitment to wounded shark skin occurs rapidly, with a marked increase in B-cells, T-cells, and unidentified leukocyte types by Day 1 post-injury. This is notably faster than in other vertebrates. For instance, in Atlantic salmon, transcriptional signatures of B- and T-cell activation typically peak around 14 days post-wounding^5^. In mammals, regulatory T-cells accumulate within 3 days, peaking at 7 days, and B-cell involvement follows a similarly delayed timeline^41,42^. In contrast, the shark data suggest a much earlier and transient immune activation, with most immune cell populations returning to near baseline by Day 14. This pattern may reflect a fast-responding immune architecture suited to the demands of a marine environment where exposure to pathogens is constant.

The observed early B-cell accumulation raises the possibility of early humoral involvement in wound defense. Although we cannot determine the immunoglobulin isotypes expressed in this dataset, sharks are known to produce several, including IgM, IgW, and IgNAR^32,33^. Indeed, we previously showed the presence of IgW and other isotypes sin spiny dogfish skin mucus^34^. Future studies that include immunoglobulin transcript or protein-level analysis will be required to determine the specific functions of B-cells in shark wound healing.

Macrophages were not identified in our dataset, which is unexpected given their established roles in mammalian and teleost wound repair^6,43^. Their absence could be due to species-specific marker differences not captured in our analysis or the presence of functionally analogous but transcriptionally distinct cell types in sharks, such as granulocytes or other myeloid cells. Alternatively, macrophages may be present at lower levels or at a different time point not captured in our sampling window.

The recovery of neural cell populations by Day 14 suggests that peripheral nerve regeneration may occur concurrently with reepithelialization in spiny dogfish. Although not directly tested, this aligns with prior studies in vertebrates showing that cutaneous nerve fibers often regenerate alongside epithelial and vascular tissues. The restoration of sensory function and trophic signaling through nerves may contribute to the coordination of wound resolution.

Interestingly, while many structural and immune components returned to baseline by Day 14, mucosal cells remained reduced, suggesting that full restoration of glandular or secretory skin features lags barrier reformation. This could reflect a functional prioritization of physical closure and immune readiness over mucus production in the early stages of healing.

## Conclusions

Experimental studies in sharks remain technically and logistically challenging. Here, we provide the first characterization of the cellular composition and morphology of spiny dogfish (*Squalus acanthias*) skin, and how it changes during wound healing. To our best knowledge it is the first time that extensive histology and snRNA-seq has been used to study shark wound healing. We show that reepithelialization occurs within 24 h, a speed comparable to warm-water species like zebrafish, and considerably faster than that reported for other cold-water teleosts such as salmonids. While scar maturation is likely to extend beyond the 35-day observation window of this study, our data indicates a structured and rapid early response.

Compared to bony fish, spiny dogfish skin contains fewer mucin-producing cells, but may have a higher baseline abundance of T-cells and B-cells. These lymphocyte populations expand markedly by Day 1 post-injury, alongside a strong infiltration of other leukocytes, suggesting a rapid and robust immune activation. Together, these findings highlight distinctive features of elasmobranch skin—including cold-tolerant reepithelialization and early adaptive immune engagement—that may offer valuable insights for comparative immunology and regenerative biology.

## Limitations

This study has several limitations. First, wounds were sampled at fixed time points, which may have missed finer dynamics of the healing process. For example, reepithelialization may have occurred faster than 24 h, but no intermediate sampling was performed between Day 0 and Day 1. Similarly, the interval between Day 1 and Day 14 may have overlooked important transitional events; this gap was based on the observation that macroscopic healing appeared slow. Second, the use of wild-caught animals housed in an artificial environment may have introduced stress-related physiological effects that influenced healing outcomes. Third, currently, approximately half of spiny dogfish genes are functionally annotated. As more species of elasmobranchii are sequenced, more novel gene networks and their corresponding cellular outputs will be identified, leading to further stratification of the cell types annotated here. Finally, the single-nucleus RNA sequencing (snRNA-seq) method used here does not capture the full transcriptomic landscape, particularly for cytoplasmic mRNAs or low-abundance transcripts, and may therefore underestimate the expression of certain genes or cell types.

## Data Availability

The datasets generated and/or analysed during the current study are available in the GEO repository (accession number GSE313154).

## Abbreviations

snRNA-seq: Single-nucleus RNA sequencing
H&E: Hematoxylin and eosin
MT: Masson's trichrome
PAS: Periodic acid–Schiff
BM: Basement membrane
MSC: Mucous-secreting cell
UMAP: Uniform Manifold Approximation and Projection
IgNAR: Immunoglobulin new antigen receptor
ECM: Extracellular matrix
dpi: Days post-injury
APC: Antigen-presenting cell
Treg: Regulatory T-cell
UV: Ultraviolet
scRNA-seq: Single-cell RNA sequencing
